# The interaction between SPARC and GRP78 interferes with ER stress signaling and potentiates apoptosis via PERK/eIF2α and IRE1α/XBP-1 in colorectal cancer

**DOI:** 10.1038/s41419-019-1687-x

**Published:** 2019-06-26

**Authors:** Yi-Jye Chern, John C. T. Wong, Grace S. W. Cheng, Angel Yu, Yaling Yin, David F. Schaeffer, Hagen F. Kennecke, Gregg Morin, Isabella T. Tai

**Affiliations:** 1 Division of Gastroenterology, Department of Medicine, University of British Columbia, Vancouver, British Columbia Canada; 2 Michael Smith Genome Sciences Center, British Columbia Cancer Agency, Vancouver, British Columbia Canada; 3 Department of Medical Genetics, British Columbia Cancer Agency, Vancouver, British Columbia Canada; 4 Cancer Surveillance & Outcomes, British Columbia Cancer Agency, Vancouver, British Columbia Canada; 5 Department of Medical Oncology, British Columbia Cancer Agency, Vancouver, British Columbia Canada; 6 Department of Pathology, University of British Columbia, Vancouver, British Columbia Canada

**Keywords:** Stress signalling, Cancer therapeutic resistance

## Abstract

Therapy-refractory disease is one of the main contributors of treatment failure in cancer. In colorectal cancer (CRC), SPARC can function as a sensitizer to conventional chemotherapy by enhancing apoptosis by interfering with the activity of Bcl-2. Here, we examine a novel mechanism by which SPARC further potentiates apoptosis via its modulation of the unfolded protein response (UPR). Using mass spectrometry to identify SPARC-associated proteins, GRP78 was identified as a protein partner for SPARC in CRC. In vitro studies conducted to assess the signaling events resulting from this interaction, included induction of ER stress with tunicamycin, 5-fluorouracil (5-FU), and irinotecan (CPT-11). We found that the interaction between GRP78 and SPARC increased during exposure to 5-FU, CPT-11, and tunicamycin, resulting in an attenuation of GRP78’s inhibition of apoptosis. In addition, we also show that SPARC can sensitize CRC cells to PERK/eIF2α and IRE1α/XBP-1 UPR signaling by interfering with ER stress following binding to GRP78, which leads to ER stress-associated cell death in CRC cells. In line with these findings, a lower expression of GRP78 relative to SPARC in CRC is associated with a lower IC_50_ for 5-FU in either sensitive or therapy-refractory CRC cells. Interestingly, this observation correlates with tissue microarray analysis of 143 human CRC, where low GRP78 to SPARC expression level was prognostic of higher survival rate (*P* = 0.01) in individuals with CRC. This study demonstrates that modulation of UPR signaling by SPARC promotes ER stress-associated death and potentiates apoptosis. This may be an effective strategy that can be combined with current treatment options to improve therapeutic efficacy in CRC.

## Introduction

Colorectal cancer afflicts more than 1.4 million individuals worldwide, and is the cause of nearly 700,000 deaths each year^1^. The emergence of therapy-refractory disease following chemotherapy continues to be a major contributor to treatment failures and the high-mortality rates observed in advanced colorectal cancer (CRC). It is now known that response to anti-cancer treatment can be influenced, in part, by the cancer cell’s response toward cellular stress induced by chemotherapeutic agents.

Endoplasmic reticulum (ER) stress signaling, or unfolded protein response (UPR), is a cellular adaptive mechanism that occurs in response to the disruption of ER homeostasis following nutrient deprivation, hypoxia, and oxidative stress^2^. Glucose-regulated protein, 78 kDa (GRP78), also referred to as Bip/HSPA5, is a well-characterized ER chaperone and also a master modulator of UPR. During cellular homeostasis, GRP78 binds to the three ER stress sensors: protein kinase RNA-like ER kinase (PERK), inositol-requiring kinase 1 (IRE1α), and activating transcription factor 6 (ATF6). However, under conditions of ER stress, GRP78 is titrated away by unfolded proteins to activate UPR and reduce cellular stress^2,3^. Activation of the UPR promotes cell survival by reducing protein influx into the ER, and the selective synthesis of the components of the protein folding^4^. However, when ER stress persists and homeostasis cannot be restored, UPR triggers cell death in cells that are beyond repair.

The upregulation of GRP78 expression has been shown to be associated with the development of chemotherapy resistance. For example, GRP78 is overexpressed in patients with castration-resistant prostate cancer^5,6^ and brain endothelial cells resistant to CPT-11 and etoposide^7^. Conversely, inhibition of GRP78 re-sensitizes B-lineage acute lymphoblastic leukemia cells that were previously refractory to vincristine^8^, and an inhibitor targeting GRP78’s ATPase domain has also been shown to resensitize breast cancer stem cells by inducing β-catenin proteasome degradation^9^. Recently, the use of HA15, a thiazole benzenesulfonamides compound specifically targeting GRP78, was able to overcome resistance to BRAF inhibitor both in vitro and in vivo in melanoma via ER stress.^10^. Therefore, a strategy that targets GRP78 may increase the efficacy of cancer treatment.

Secreted protein, acidic and rich in cysteine (SPARC), appears to have opposing effects to GRP78, in that it functions as a chemo-sensitizer in certain cancers, such as CRC^11–13^, hepatocellular carcinoma^14^, pancreatic cancer^15^, and osteosarcoma^16^. SPARC has been demonstrated to suppress cell cycle progression in both ovarian carcinoma^17^ and acute myeloid leukemia^18^. Loss of SPARC is associated with an accumulation of reactive oxygen species and urothelial cell proliferation in bladder cancer^19^. In CRC, apoptosis can be induced in the presence of significantly lower concentrations of chemotherapy when SPARC is overexpressed^11^, by potentiating the activation of the extrinsic cascade of apoptosis via its interaction with caspase 8, with subsequent involvement of the intrinsic cascade, via Bid^12,13^.

The mechanisms by which SPARC influences cancer cells’ response to chemotherapy appears complex. In this study, we further examine its role by demonstrating that its interaction with GRP78 promotes CRC cells to undergo ER stress-associated death when exposed to chemotherapy. We further demonstrate that in primary CRC tissues, the relative expression of GRP78 to SPARC can be prognostic of progression-free survival in individuals with advanced CRC.

## Results

### A novel interaction between SPARC and GRP78 occurs in CRC

In order to help further understand the mechanisms by which SPARC may facilitate apoptosis in response to chemotherapy, we began by identifying potential SPARC-interacting partners. We used the C-terminal V5 epitope-tagged SPARC expressed in MIP101 cells to co-immunoprecipitate (co-IP) SPARC-interacting protein complexes. Following co-IP, the complexes were digested with trypsin and subsequently processed for tandem mass spectrometry (MS/MS) analysis. To exclude nonspecific binding proteins, empty vector-transfected MIP/ZEO cells were used as co-IP control. Glucose-regulated protein 78 kDa (GRP78) was identified with high peptide coverage as a putative SPARC-interacting protein (Table 1).

**Table 1:** Immunoprecipitation- mass spectrometry identification of SPARC-interacting proteins

The MS identification scores for SPARC and candidate interacting proteins are shown							
Bait protein	X!Tandem			Mascot			Accession
	Log(E)^a^	# peptides	% coverage	Score^b^	# peptides	% coverage	UniPROT
SPARC Secreted protein acidic and rich in cysteine (C-term V5 epitope tag)	−56	8	22	310	11	33	P09486
*Interacting protein*							
GRP78 78 kDa glucose-regulated protein	−86	11	25	484	22	39	P11021

^a^The log(E) value is an estimate of the probability that the protein assignment occurred randomly, as calculated by the X!Tandem algorithm against the human UniPROT protein database

^b^Mascot score, as calculated by the Mascot algorithm against the human UniPROT protein database

**Fig. 1:**
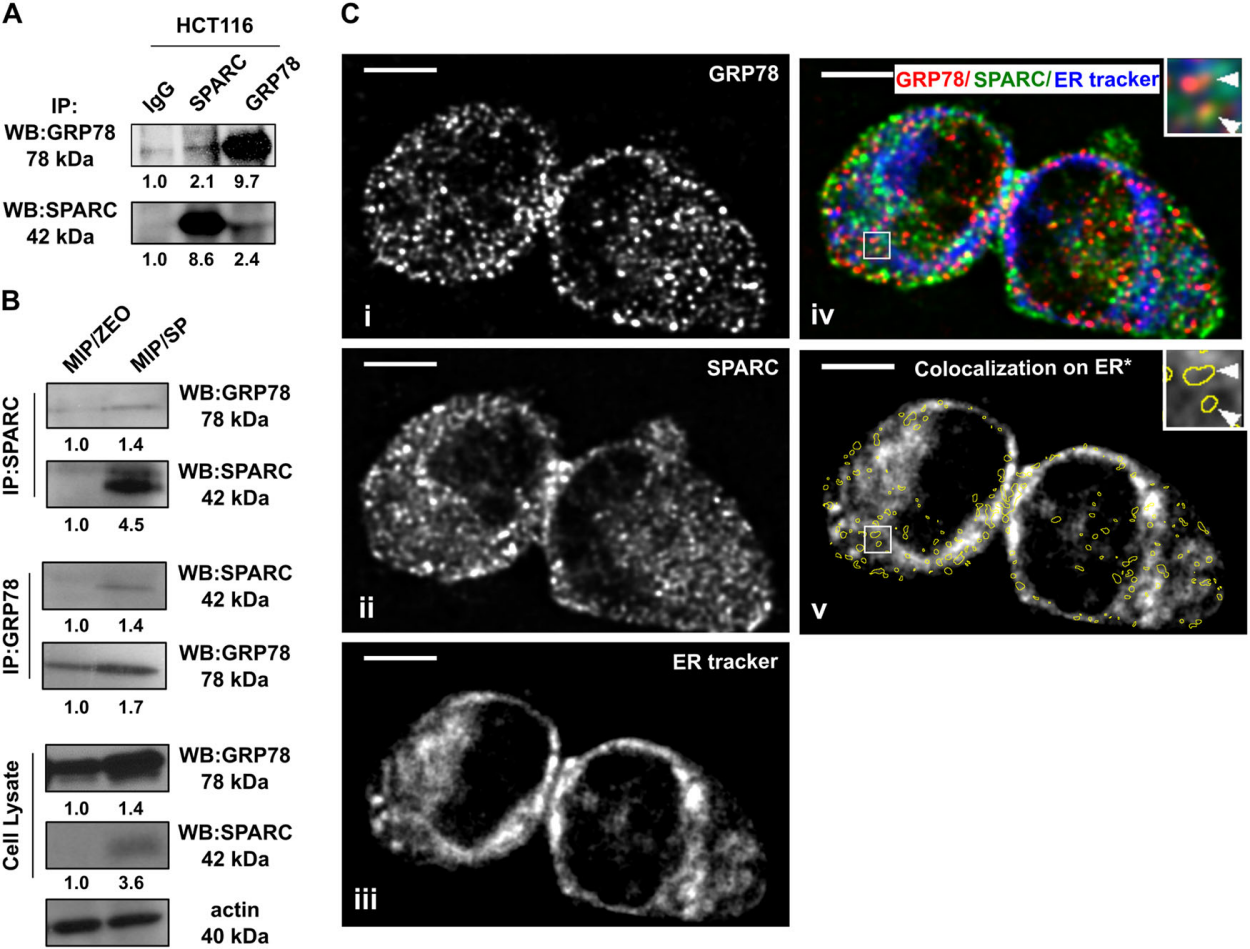
Interaction between GRP78 and SPARC in CRC. Co-immunoprecipitation of SPARC with GRP78 in (**a**) HCT116 and confirmed in (**b**) MIP/Zeo and MIP/SP cells. (**c**) Colocalization of (i) GRP78 and (ii) SPARC in HCT116 by confocal microscopy and immunofluorescent analysis. (iii) The endoplasmic reticulum was stained with ER tracker blue-white DPX dye. (iv) Images were overlapped, and (v) the GRP78:SPARC colocalization pixels were identified by Colocalization Finder plugin in ImageJ and highlighted with yellow circles. The average ER tracker staining intensity in the highlighted areas was calculated to determine the percentage of GRP78:SPARC colocalization in the ER. Scale bar = 5 μm

In order to confirm the interaction between GRP78 and SPARC, co-IP analysis using intrinsically high SPARC-expressing human CRC cell line HCT116 and SPARC-overexpressing MIP/SP was conducted. The results confirmed the presence of an interaction between GRP78 and SPARC in endogenous-SPARC-expressing HCT116 (Fig. 1a) and SPARC-overexpressing MIP/SP, but not MIP/Zeo cells. (Fig. 1b). In addition, confocal microscopic immunofluorescent analysis also suggested colocalization of GRP78 (Fig. 1c–i) with SPARC (Fig. 1c-ii) in HCT116. Given GRP78’s role as an ER chaperone, it was interesting to note that the majority of the colocalization pixels demonstrating the interaction between GRP78 and SPARC overlapped with the ER tracker-labeled regions (65.4 ± 4.5%, *N* = 3) (Fig. 1c-iii), suggesting that the interaction between GRP78 and SPARC may be occurring predominantly in the ER (Fig. 1c-iv, v).

### A dynamic interaction between SPARC and GRP78 in the ER occurs in the presence of ER stress

**Fig. 2:**
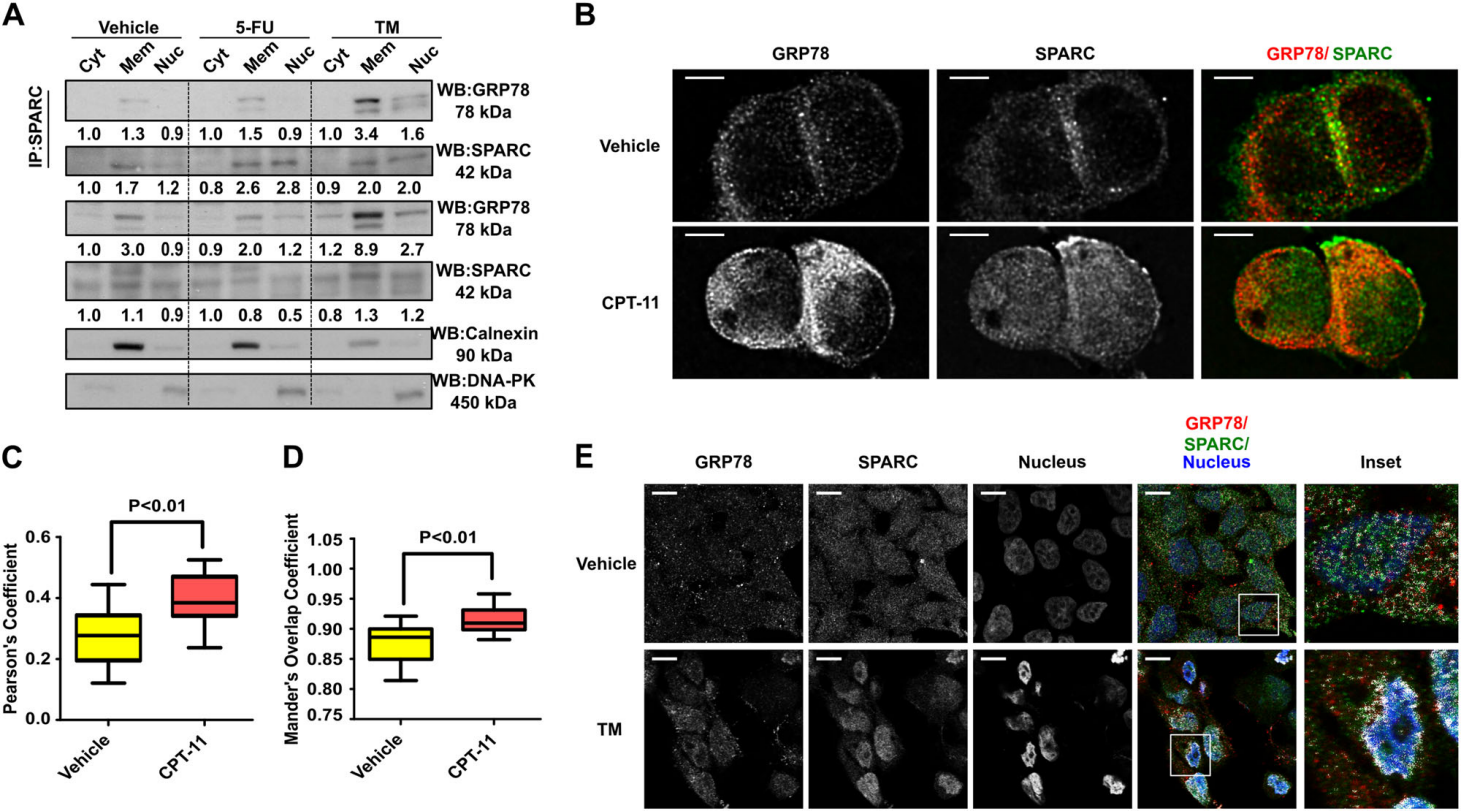
Dynamic interaction between SPARC and GRP78 in the ER occurs with chemotherapy-induced ER stress. (**a**) HCT116 were treated with 5-FU (25 μM, 12 h) and TM (1 μg/ml, 6 h) followed by cell fractionation, co-IP and Western blot analysis of cytoplasmic (Cyt), membranous (Mem), and nuclear (Nuc) fractions. Calnexin and DNA-PK served as quality control of cell fractionation. Densitometry quantification was performed, and the densitometry values were indicated below the blots. (**b**) Immunofluorescent analysis of HCT116 cells treated with CPT-11 (50 μM, 12 h) or vehicle by confocal microscopy. Cells were labeled with primary antibodies against SPARC (green) and GRP78 (red). Quantitative colocalization analysis was performed followed by deconvolution of the images. Scale bar = 5 μm. (**c**) The Pearson’s coefficient and (**d**) the overlap coefficient was calculated from 12 images (*N* = 12) for each group. (**e**) Immunofluorescent analysis of MIP/SP cells treated with TM (1 μg/ml, 6 h) or vehicle. Colocalization of GRP78 and SPARC was evident in TM-treated cells based on the analysis using the ImageJ Colocalization Finder plugin (white). Fluorescence intensities were gamma-corrected (gamma = 2.0) to reveal the colocalization pixels in the perinuclear region. Scale bar = 10 μm

Based on the confocal microscopy findings suggesting that the interaction between GRP78 and SPARC occurs predominantly in the ER, we proceeded to specifically examine this possibility by co-IP/immunoblot analysis. Given GRP78’s known ER-stress modulatory function, we were also interested in examining whether the interaction between GRP78/SPARC could be influenced by the level of ER stress in CRC cells. Co-IP assays were performed following cellular fractionation of endogenous-SPARC-expressing HCT116 cells that were treated with either 25 µM 5-FU or 1 µg/ml TM. The interaction between endogenous GRP78 and SPARC was mainly observed in the ER-containing microsomal fractions (Fig. 2a, similar fraction that contained ER-specific calnexin). Interestingly, this interaction increased following ER-stress induced by 5FU and TM, predominantly within the microsomal fraction, and to a lesser extent, in the nuclear fraction (Fig. 2a, DNA-PK-positive fraction). We also characterized this interaction, using another chemotherapy, CPT-11, and noticed a similar increase in the interaction between GRP78 and SPARC in the microsomal fractions (Supplementary Fig. 1A). These observations led us to believe that the interaction between SPARC and GRP78 is augmented in the setting of ER-stress.

The interaction that occurs between GRP78 and SPARC in response to ER stress was also further evaluated by confocal microscopy. In HCT116 cells, a greater concentration of colocalized pixels were seen following induction of ER stress with CPT-11 exposure (Fig. 2b, c, d). Similarly, MIP/SP cells exposed to ER stressor tunicamycin (TM) resulted in an increase in the interaction between these two proteins in the perinuclear region (as indicated by an increase in colocalized pixels, Fig. 2e). Quantitative analysis demonstrated that following CPT-11 treatment, a statistically larger percentage of SPARC and GRP78 signals overlapped as represented by higher Pearson’s (by R_r_ = 0.265 ± 0.028 vs. R_r_ = 0.392 ± 0.024, *P* < 0.01) and Mander’s overlap coefficient (R = 0.877 ± 0.010 vs. R = 0.915 ± 0.006, *P* < 0.01) (Fig. 2c, d). This indicates that a dynamic interaction between GRP78 and SPARC exists that is dictated by changes in cellular stress (for example, following exposure to chemotherapy).

### GRP78 reduces SPARC’s proapoptotic effects during ER stress

**Fig. 3:**
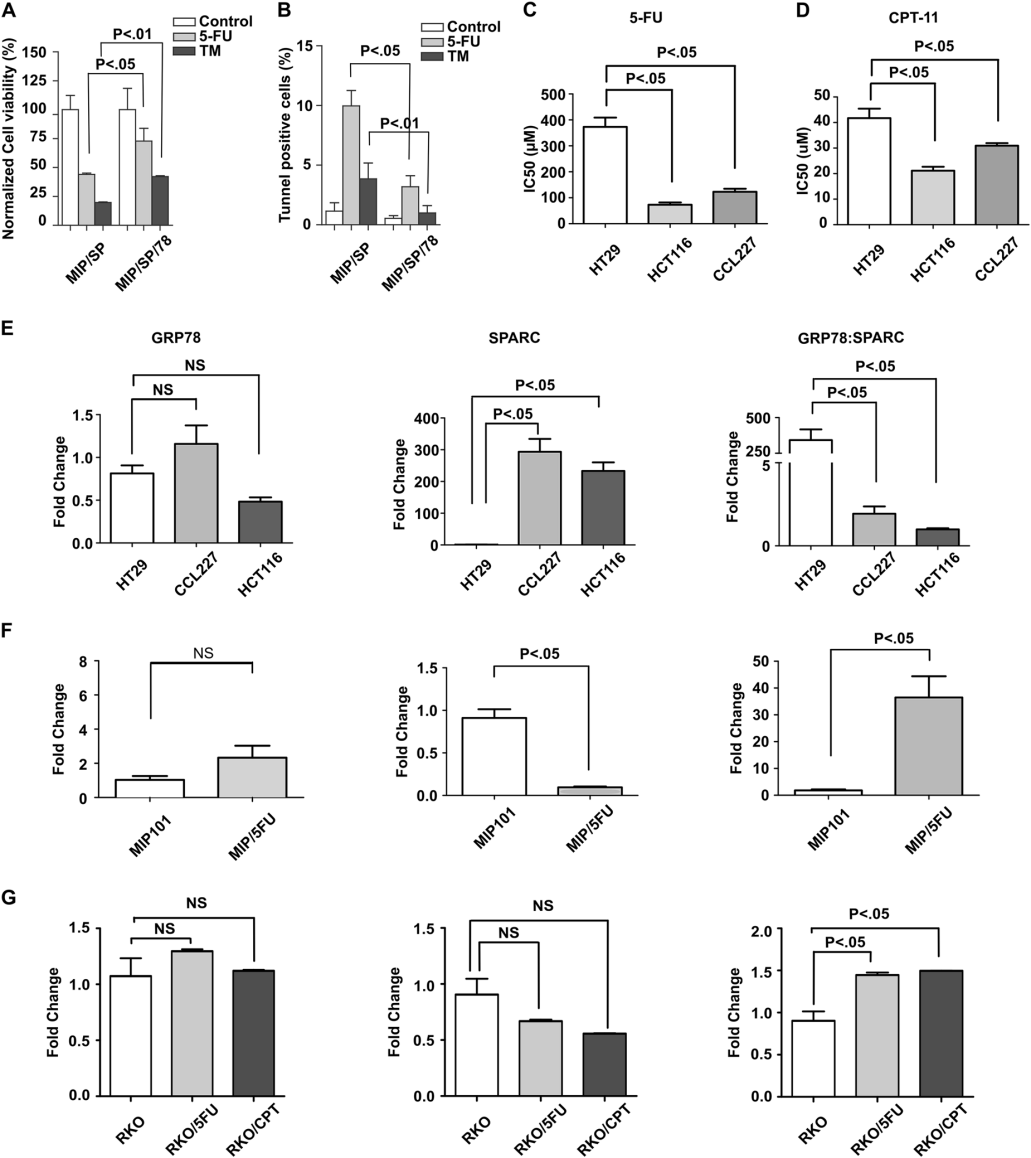
SPARC attenuates GRP78’s pro-survival effects under ER stress. The effects of SPARC and GRP78 expression on cell viability under ER stress were determined by using MIP/SP and GRP78-overexpressing MIP/SP cells (MIP/SP/78) following exposure to 5-FU (5 μM) and TM (1 μg/ml) for 48 h. Cell viability and apoptosis were analyzed by (**a**) MTS and (**b**) TUNEL assays, respectively. Cell viability and IC_50_ of CRC cell lines HT29, CCL227, and HCT116 following exposure to (**c**) 5-FU and (**d**) CPT-11 were determined by MTS assay. The mRNA expression levels of GRP78 (left), SPARC (middle), and the relative expression level (GRP78:SPARC) (right) in (**e**) CRC cell lines (HT29, CCL227, HCT116), (**f**) MIP101, and (**g**) RKO 5-FU- and CPT-11-resistant cell lines were determined by q-RT-PCR. (The data represent mean ± SEM, *N* = 3)

Based on the above observations that the interaction between GRP78 and SPARC increased under conditions of cellular ER stress (as in the presence of chemotherapy), we wondered if changes in the expression level of the two proteins would not only alter their protein interaction but also influence cell viability following exposure to ER-stress, including chemotherapy. To answer this question, we modulated GRP78 expression level in the SPARC-overexpressing MIP/SP cells and evaluated the viability of MIP/SP and MIP/SP/78 cells (MIP101 cells co-expressing SPARC and GRP78) following exposure to TM and 5-FU. The overexpression of GRP78 in MIP/SP cells increased cell viability despite exposure to 5-FU and TM: cell viability increased from less than 50% in MIP/SP following 5-FU exposure to more than 60% in MIP/SP/78 cells. A similar trend was also observed following tunicamycin exposure (Fig. 3a). This is also represented by a reduction in TUNEL-positive apoptotic MIP/SP/78 in comparison with MIP/SP following treatment of 5-FU and tunicamycin (Fig. 3b). These results suggest that the expression of GRP78 may reduce SPARC’s ability to promote ER stress-associated death in CRC cells following chemotherapy. Furthermore, it demonstrates that it is the relative expression level of GRP78 to SPARC that influences the survival of cancer cells in response to chemotherapy.

To confirm that the relative expression of GRP78:SPARC is a better determinant of cell viability in CRC, several CRC cell lines were examined, with the corresponding IC_50_ values of 5-FU and CPT-11 determined by MTS assays. No correlation was observed between GRP78 expression level (Fig. 3e, left panel) and the IC_50_ for 5-FU (Fig. 3c) and CPT-11 (Fig. 3d) in the CRC cells (5-FU: HT29, 373.31 ± 61.44 µM; CCL227, 123.19 ± 19.85 µM; HCT116, 73.02 ± 15.20 µM. CPT-11: HT29, 41.73 ± 6.42 µM; CCL227, 30.94 ± 1.70 µM; HCT116, 21.13 ± 2.69 µM).

However, SPARC expression level was found to be the lowest in the most resistant HT29, although its expression is higher in the CCL227 compared with the more sensitive HCT116 cells. (Fig. 3e, middle panel). Again, we observed that the relative expression level of GRP78 to SPARC may be a better indicator of CRC cells’ response toward chemotherapy: while the most resistant HT29 has the highest ratio of GRP78 to SPARC expression level, the most sensitive HCT116 also shows the lowest relative expression level among the three (Fig. 3e, right panel). We also observed similar results in our MIP 101 and RKO 5-FU and CPT-11-resistant cell lines (Fig. 3f, g). This interesting finding suggests that the ratio of GRP78 to SPARC expression may be a better indicator of a cell’s ability to survive following exposure to chemotherapy, and consequently, a better predictor of resistance to therapy. This potential observation motivated us to examine whether the ratio of GRP78 to SPARC expression level could serve as a prognostic biomarker for patients with CRC (see below).

### Low expression of GRP78 to SPARC ratio correlates with improved survival in individuals with CRC

**Fig. 4:**
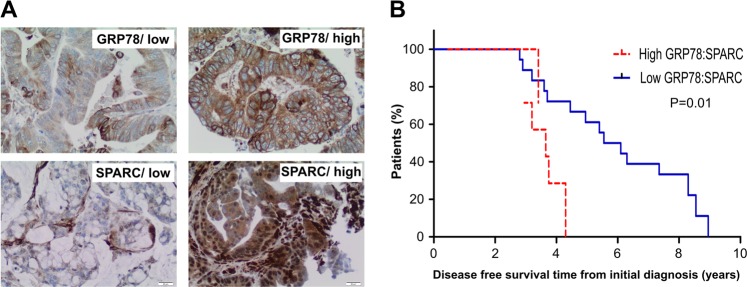
CRC patients with a low ratio of GRP78:SPARC protein expression have improved prognosis. (**a**) Representative images of GRP78 and SPARC expression in CRC scored as high expression and low expression; x20, magnification. (**b**) Kaplan–Meier survival curves of patients with CRC. Individuals with tumor expressing low GRP78:SPARC ratio have significantly higher median disease-free survival

Using a tissue microarray (TMA) containing CRC tissues of 143 individuals with CRC, the levels of expression of GRP78 and SPARC in CRC tissues were analyzed to determine if there is any correlation between the expression of these individual proteins and disease-free survival in individuals with CRC (Fig. 4a). There was no significant statistical association between the expression level of either GRP78 or SPARC with disease-free survival (data not shown). However, when the relative expression of GRP78 to SPARC was examined, low expression of GRP78/SPARC was associated with a significantly better prognosis: the median disease-free survival of 5.50 months (95% CI: 3.59, 7.45) in comparison with those whose CRC had high GRP78/SPARC expression, median disease-free survival of 3.71 (95% CI: 2.93, 4.30; *p*-value = 0.01) (Fig. 4b). This result suggests that the low expression of GRP78 to SPARC ratio is associated with improved survival in CRC patients, and may potentially be a prognostic marker for CRC.

### SPARC sensitizes CRCs to PERK/eIF2α and IRE1α/XBP-1 ER stress signaling

**Fig. 5:**
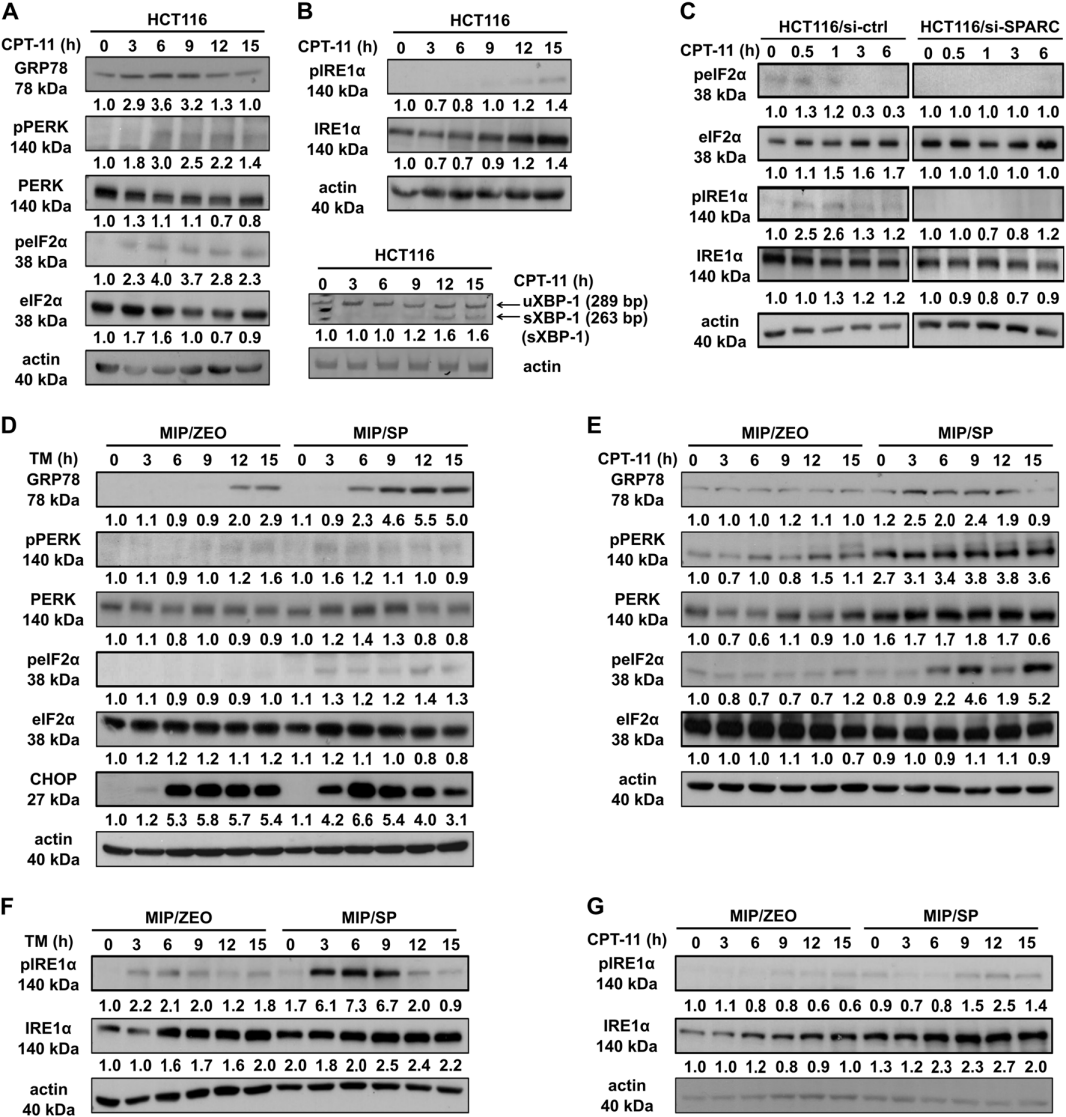
SPARC promotes early activation of PERK-eIF2α and IRE1α-sXBP ER stress signaling in endogenous HCT116 and MIP/SP cells. HCT116 treated with CPT-11 (50 μM) for various time intervals were analyzed for the presence of the activation of (**a**) PERK/eIF2α and (**b**) IRE1α/XBP-1. The spliced form of XBP-1 was detected by RT-PCR analysis. (**c**) Western blot analysis of ER stress signaling in HCT116 cells following SPARC siRNA knockdown and exposure to CPT-11 (50 μM). Activation of (**d**, **e**) PERK/eIF2α and (**f**, **g**) IRE1α/XBP-1 signaling in MIP/Zeo and MIP/SP following treatment with TM (1 μg/ml) or CPT-11 (50 μM) were examined by Western blot analysis

Since GRP78 is a known modulator of ER stress signaling, we next examined the activation of ER stress signaling in the presence of SPARC to understand the mechanism behind the effects of the interaction between GRP78 and SPARC following ER-stress, including chemotherapy. We found that CPT-11 is able to induce ER stress (as shown by an upregulation of GRP78 expression), and activate both PERK/eIF2α, as demonstrated by the phosphorylation of PERK (Ser713), eukaryotic initiation factor 2-alpha (eIF2α, Ser51), and the induction of activating transcription factor 4 (ATF4), the downstream transcription factor of phopho-eIF2α (Fig. 5a; supplementary Fig. 1B). It also activated IRE1α/XBP-1 ER stress signaling, as shown by the phosphorylation of IRE1α (S724) and the alternative splicing of XBP-1 in HCT116 cells (Fig. 5b). In line with these observations, the knockdown of SPARC with short-interfering RNA attenuated the activation of both PERK/eIF2α and IRE1α/XBP-1 signaling (Fig. 5c).

To further confirm SPARC’s ability to influence ER stress signaling, MIP/Zeo and SPARC-overexpressing MIP/SP cells were used to examine these effects of ER-stress signaling following exposure to TM and CPT-11. We observed that in MIP/SP cells, GRP78, phospho-PERK, phospho-eIF2α, and ATF4 were detected at earlier time points than in MIP/Zeo following exposure to TM and CPT-11 (Fig. 5d, e; Supplementary Fig. 1B). Importantly, C/EBP-homologous protein (CHOP), a transcription factor that mediates ER-initiated apoptotic cell death, was also induced earlier in MIP/SP treated with TM and CPT-11 (Fig. 5d; Supplementary Fig. 1C). Similarly, phospho- IRE1α was also more prominently expressed in MIP/SP following exposure to tunicamycin and CPT-11 (Fig. 5f, g). Moreover, we also examined the apoptotic proteins and found that the expression of the anti-apoptotic protein Bcl-2 is decreased while the expression of the proapoptotic Bax increased in MIP/SP, indicating a stronger induction of apoptosis in the SPARC-overexpressing cells under the drug treatment (Supplementary Fig. 1D, E). Notably, SPARC expression seems to also influence autophagic response as shown by the increased conversion of microtubule-associated protein light chain 3 (LC3) (LC3-I to LC3-II), although the level of Beclin-1 remains unchanged (Supplementary Fig. 1D, E). Importantly, similar observations of earlier ER stress signaling and apoptosis induction were also observed in MIP/SP exposed to 5-FU (Supplementary Fig. 2). Overall, these data indicate that SPARC may restrict the capacity of CRC cells to sustain ER homeostasis, rendering CRC cells more sensitive to stress stimulus, resulting in earlier activation of ER stress signaling and the downstream apoptotic cascade.

### SPARC modulates ER stress signaling through its interference in the binding between ER stress sensor and GRP78

**Fig. 6:**
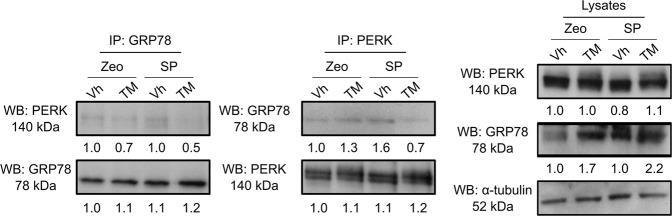
SPARC interferes with the binding between GRP78 and PERK under ER stress. MIP/Zeo and MIP/SP were treated with TM (1 μg/ml, 6 h) and the cell lysates were immunoprecipitated with anti-GRP78 and anti-PERK antibodies, respectively, followed by Western blot analysis

**Fig. 7:**
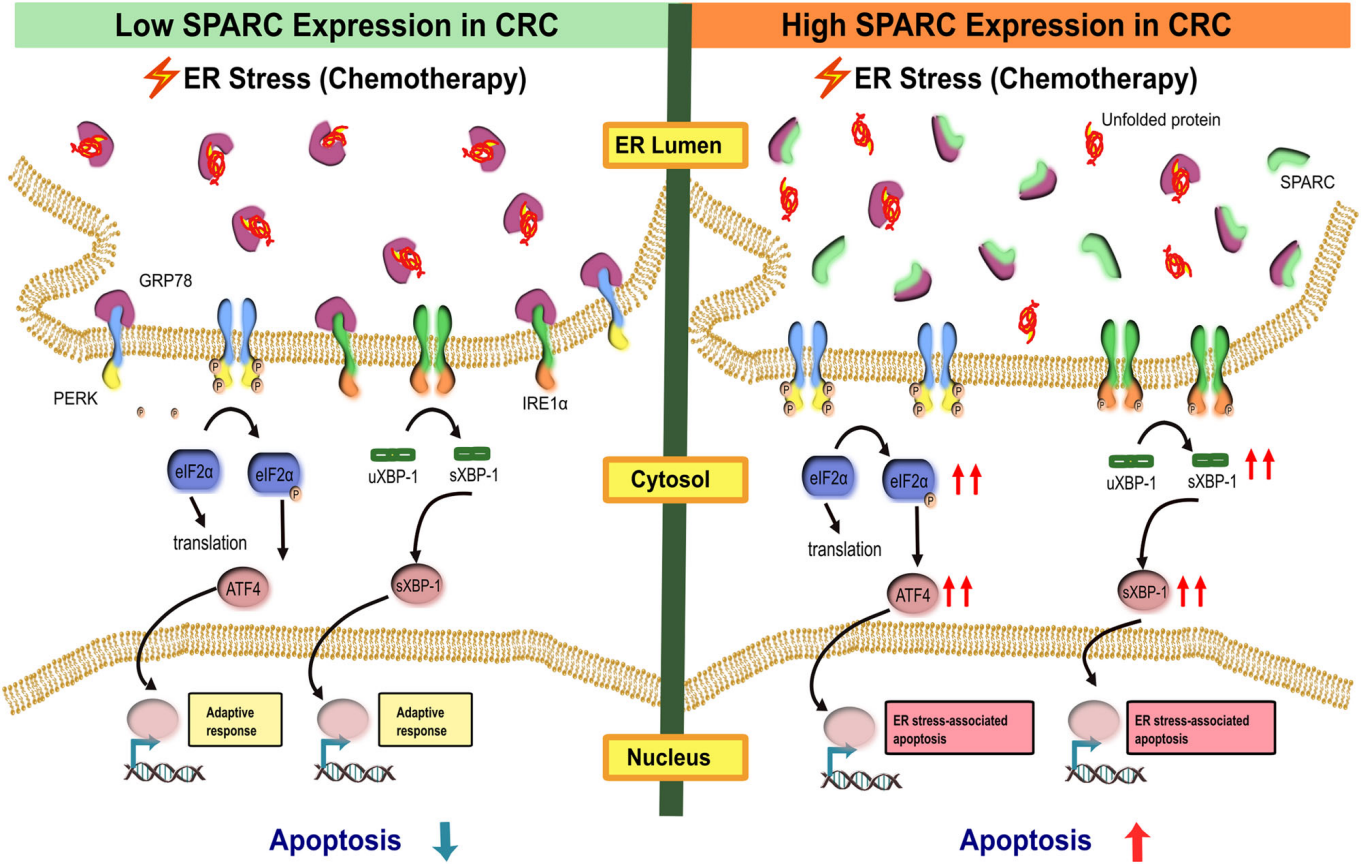
SPARC reduces the tolerance to ER stress in CRC cells. SPARC interferes with the binding between GRP78 and ER stress sensors (such as PERK and IRE1α) via its interaction with GRP78. Under ER stress, the stress stimulus is amplified in high SPARC-expressing cells, as SPARC facilitates the dissociation of GRP78 from the ER stress sensors, thereby lowering the threshold of ER stress signaling and subsequent activation of the apoptotic cascade

Under conditions of cellular stress, the dissociation of GRP78 from ER stress sensors leads to the activation of ER stress signaling. We hypothesized that SPARC may sensitize the cells to the activation of ER stress signaling by interfering with the binding between GRP78 and ER stress sensors. To test this hypothesis, we conducted co-IP assays using MIP/Zeo and MIP/SP cells exposed to TM to examine the binding between GRP78 and ER stressors in the presence of SPARC, while under ER stress. We noticed that the level of interaction between GRP78 and PERK was decreased in the MIP/SP cells following exposure to TM (Fig. 6). Conversely, this interaction only slightly decreased in MIP/ZEO cells following tunicamycin treatment. Similar results were also found in the cells treated with CPT-11 (Supplementary Fig. 1F). These results suggest that SPARC may weaken the binding between GRP78 and PERK, thereby lowering the threshold of ER stress signaling activation under stress and facilitate ER-stress-associated cell death (Fig. 7).

## Discussion

In this study, we demonstrated that SPARC sensitizes CRC cells to chemotherapy by modulating ER stress signaling to promote ER stress-associated cell death. We identified a novel interaction between SPARC and GRP78 and found that this interaction increased during treatment with chemotherapy agents and ER-stress inducers. In CRC cells exhibiting an overexpression of SPARC, ER stress signaling was enhanced, as evidenced by an earlier activation of PERK/eIF2α- and IRE1α/XBP-1-mediated signaling following exposure to 5-FU, CPT-11, and tunicamycin. Interestingly, while higher expression of GRP78 in CRC has been shown to contribute to chemoresistance, we were able to demonstrate that SPARC may counteract this effect. In fact, the relative expression level of GRP78 to SPARC in CRC cells may determine the level of chemosensitivity of cancer cells to drug therapy. In support of this observation, we also noted that tissues from CRC patients that demonstrated a higher GRP78:SPARC expression ratio had a lower overall survival. The findings of this study identify SPARC as a novel modulator of ER stress, and further defines its role in enhancing response to drug therapy in CRC.

ER stress signaling is often activated in cancer in order to support the rapid growth of cancer cells in a challenging environment that would be inadequate to support the viability of normal cells^20,21^. However, this signaling event can be viewed as a double-edged sword: it can support cancer cell survival in an adverse environment while promoting cell death under harsh and sustained stress conditions. Mechanisms determining the switch from adaptive to cell death signaling under ER stress have not yet been clearly defined, and the outcome of the activation of ER stress signaling may depend on the type, intensity, and duration of the stimulus in relation to active changes in cellular environment. For example, under mildly stressful cellular conditions, the PERK-eIF2α-ATF4 axis may facilitate the expression of genes involved in amino acid transport and glutathione biosynthesis, which facilitates the reestablishment of protein homeostasis in order to support cellular survival^22,23^. However, under conditions of severe cellular stress (e.g., exposure to chemotherapy), PERK signaling can induce the expression of CHOP, which promotes apoptosis by upregulating the expression of proapoptotic BH3-only proteins while suppressing Bcl-2^23,24^. In this study, we show that SPARC serves as a modulator of ER stress: in cancer cells with an abundance of SPARC, this protein lowers the threshold of ER stress signaling activation by interfering with the binding between GRP78 and stress sensors. Similar to other proteins that can regulate the magnitude of ER stress signaling, such as BAX, BAK^25^, and ASK1-interacting protein 1 (AIP1)^26^, SPARC’s known association and promotion of apoptosis^12^, suggest an important association between apoptosis and ER stress signaling in determining cellular events in cancer.

The involvement of SPARC in ER stress signaling does not appear to be restricted to CRC. For instance, in gliomas, SPARC mRNA undergoes endonucleolytic cleavage by IRE1α in response to ER stress^27^, and the loss of functional IRE1α leads to an upregulation of SPARC^28^. In this study, we showed that the expression of SPARC in CRC promotes the activation of PERK and IRE1α signaling. This suggests that the cleavage of SPARC by activated IRE1α may serve as a negative feedback mechanism that reduces SPARC level to attenuate the activation of ER stress signaling. We also began an initial assessment of autophagy in relation to ER stress and noticed a greater susceptibility of cells overexpressing SPARC to undergo autophagy following ER-stress induced by chemotherapy. Our observation is supported by previous studies that demonstrated autophagy-mediated apoptosis following radiation therapy-mediated ER stress in neuroblastomas overexpressing SPARC^29^. This is not surprising, as the induction of autophagy, similar to ER stress, can either lead to cell death or survival depending on the cellular and/or environmental context^30^. Our findings suggest that SPARC may play a key role in coordinating ER stress response, and autophagy signaling in cancer cells to facilitate a more favorable response to chemotherapy^31,32^.

In contrast to SPARC, GRP78, which is often upregulated on the cell surface in malignancy, can interact with PI3K, CRIPTO, IGF1-R in the breast, prostate, and hepatoma cells, respectively, to promote oncogenic events^33–37^. It is therefore not surprising that the use of an antibody against cell surface GRP78 has been shown to supress cell survival in chemotherapy-resistant multiple myeloma and glioma cells^33,38^. Our demonstration that SPARC, by potentiating ER stress signaling through its interaction with GRP78 to promote cell death following chemotherapy, may be another mechanism that likely contributed to the dramatic regressions of therapy-resistant CRC tumor xenografts following SPARC-based therapy in previous reports^11,13^. Therefore, a potential strategy in the treatment of CRC may involve the modulation of ER-stress using a combination of SPARC-based and anti-GRP78-based therapies in a more personalized approach. This would tailor the treatment to individuals with CRC that have high GRP78 but low SPARC expression, in a more personalized manner. This sub-population of individuals would most likely benefit from this tailored treatment, since our findings also suggest that individuals with CRC expressing relatively higher GRP78:SPARC have poorer overall survival. An approach that allows SPARC- and anti-GRP78-based therapies to modulate ER stress in high GRP78:SPARC-expressing tumors, would be effective in heterogenous tumors across many different mutational profiles, and lead to more favorable outcomes. Another advantage of this SPARC-based therapy is that it induces cell death through alternative mechanisms that can complement current conventional-based chemotherapies and monoclonal antibody-based target therapies in CRC. The inclusion of SPARC and anti-GRP78-based therapies would likely allow a dose reduction in conventional therapies which would minimize dose-limiting adverse effects thereby resulting in superior outcomes.

Our findings also indicate that the expression of GRP78:SPARC may be an effective prognostic biomarker in CRC. Prognostic and predictive biomarkers that can help identify individuals at higher risk of early relapse and help guide treatment options are greatly needed. SPARC expression has previously been assessed for its utility as a prognostic biomarker, and in line with current observations, high SPARC expression in primary CRC has been shown to be associated with better outcomes, based on longer disease-free survival in stage II and III CRC patients^39^. However, the converse has also been shown, as upregulation of SPARC has also been linked to poor outcomes following adjuvant chemotherapy in CRC patients^40^. Expression of GRP78 in CRC and its utility as a biomarker has also encountered variable results, as high GRP78 expression has been associated with both good and poor prognosis in CRC patients following chemotherapy^41,42^. These studies demonstrating paradoxical findings indicate that single-gene markers may not be sufficient to predict chemotherapeutic response, given the complex signaling networks in CRC. Consistent with this notion, we found that while no association was in relation to SPARC or GRP78 expression level and the disease-free survival, the relative expression level of the ratio of GRP78 to SPARC, could influence the clinical outcome for individuals with CRC.

In summary, this study demonstrates that SPARC modulates ER stress signaling through its interaction with GRP78. Via this mechanism, SPARC further potentiates apoptosis during chemotherapy treatment in CRC by inducing ER stress-associated cell death. Our findings can lead to alternative strategies that can help guide the management of individuals with CRC.

## Materials and methods

### Cell lines

Human CRC cell lines MIP101, MIP101 cells resistant to 5FU (MIP/5FU), MIP101 cells overexpressing SPARC (MIP/SP) and empty vector control (MIP/ZEO), RKO and RKO cells resistant to 5FU (MIP/5FU), and CPT-11 (RKO/CPT) were used. Cells were maintained in the Dulbecco’s Modified Eagle Medium (DMEM) (Invitrogen) supplemented with 10% newborn calf serum (NCS) and 1% penicillin–streptomycin and 1% kanamycin (Invitrogen) at 37 °C in a humidified atmosphere with 5% CO_2_. For MIP/SP and MIP/ZEO cells, DMEM was also supplemented with 0.1% Zeocin (Invitrogen). Resistant cells MIP/5FU, RKO/5FU, and RKO/CPT were supplemented with 0.2 mM 5FU, 10 μM 5FU, or 70 μM CPT-11, respectively.

### Mass spectrometry analysis

A C-terminal V5 epitope tagged SPARC cDNA vector was transiently expressed in MIP101 cells. SPARC protein complexes were immunoprecipitated with anti-V5 antibody (Applied Biomaterials Inc.) and eluted with elution buffer (V5 peptide 400 μg/ml, ammonium bicarbonate 50 mM). Proteins immunoprecipitated with an empty vector transfection served as controls. The samples were run on 4–12% precast NuPAGE gels (Invitrogen) and each lane was cut into 16 horizontal slices. The gel slices were processed for tandem mass spectrometry analysis using in-gel dehydration, alkylation, trypsin digestion, and extraction^43^. The peptides were analyzed by HPLC-electrospray-tandem mass spectroscopy (ESI-MS/MS) on a 4000 QTrap mass spectrometer (Applied Biosystems/Sciex, Foster City, CA, USA) using standard procedures^43^. The MS/MS spectra emanating from the gel slices for each lane were concatenated and searched against the UniProt human database using the X!Tandem (http://www.thegpm.org/tandem) and Mascot (Matrix Science, Boston, MA, USA) search algorithms. Candidate interacting proteins (Table 1) were those that were observed with two or more peptides and not in the control sample.

### qRT-PCR

Cells were seeded in 60 -mm dishes and were harvested at 80% confluency. The total RNA were extracted with Trizol (Invitrogen) according to the manufacturer’s instruction. Oligo(dT)-primed first-strand cDNA was synthesized from 1 μg of RNA by using Reverted H Minus Reverse Transcriptase (Thermo) following the manufacturer’s instruction. The following primers were used for qRT-PCR: RPL19: 5′-TGAAATCGCCAATGCCAACTC-3′ (sense) and 5′-GGCTGTACCCTTCCGCTTACC-3′ (antisense); GRP78: 5′-GGCCGAGGAGGAGGACAAGA-3′ (sense) and 5′-GGCGGCATCGCCAATCAGAC-3′ (antisense); and SPARC: 5′-CCCTGTACACTGGCAGTTCG-3′ (sense) and 5′-CCAGGGCGATGTACTTGTCA-3′ (antisense). The transcript levels of RPL19 were used for normalization. qRT-PCR was performed by using KAPA SYBR FAST qPCR kit (KAPA Biosystems) in ABI Prism 7900HT Sequence Detection System (Applied Biosystems).

### RT-PCR assay for *XBP-1* splicing detection

The total RNA preparation and cDNA synthesis were processed as described above, followed by PCR with Platinum^®^ Taq DNA Polymerase High Fidelity (Invitrogen) using primers flanking the splice site^44^. The primer sequences for sXBP-1 detection were: XBP1: 5′-TTACGAGAGAAAACTCATGGCC-3′ (sense) and 5′-GGGTCCAAGTTGTCCAGAATGC-3′ (antisense); β-actin: GCCACGGCTGCTTCC-3′ (sense) and 5′-GGCGTACAGGTCTTTGC-3′ (antisense). The PCR condition was 94 °C for 2 min, followed by 94 °C for 30 s, 58 °C for 30 s and 68 °C for 30 s for 27 cycles. Unspliced XBP-1 gave a product of 289 bp, and the spliced cDNA of 263 bp. PCR products were separated by 5% urea denaturing PAGE followed by ethidium bromide staining and quantification by ImageJ (National Institute of Health, USA).

### Cellular fractionation and immunoprecipitation

MIP/SP and HCT116 cells were seeded into 100 -mm dish overnight. MIP/SP cells were lysed in lysis buffer [25 mM Tris-HCl (pH 7.4), 150 mM NaCl, 1% NP-40 and 5% glycerol]. HCT116 cells were subjected to modified cell fractionation protocol^45^ after treatment with 5-FU or tunicamycin (cat. 654380, Millipore). The treatment duration with TM and 5FU were different, as these were based on preliminary experiments that indicated that TM induction of ER stress occurred at earlier time points than 5-FU. HCT116 cells were lysed in Buffer A [10 mM HEPES–KOH (pH 7.9), 1.5 mM MgCl_2_, 120 mM KCl, and 0.2 mM PMSF] and incubated for 10 min. Samples were centrifuged at 14,000 rpm for 1 min to collect the cytosolic fraction. The cell pellets were resuspended in Buffer C [20 mM HEPES–KOH (pH 7.9), 25% glycerol, 420 mM NaCl, 1.5 mM MgCl_2_, 0.2 mM EDTA, and 0.2 mM PMSF] and incubated for 20 min. After centrifugation at 14,000 rpm for 2 min, the supernatant was collected as nuclear fractions, and the pellets were resuspended in IP lysis buffer and incubated for 30 min. Samples were centrifuged 14,000 rpm for 10 min and the supernatant containing membrane fractions were collected. The experiments were conducted at 4 °C and all buffers were supplemented with 1% proteinase inhibitor cocktail (Sigma). One milligram of MIP/SP cell lysate was incubated with 5 μg of mouse anti-human SPARC antibodies (HTI, AON-5031), rabbit anti-human GRP78 antibody (Santa Cruz, sc-13968), or rabbit anti-PERK antibody (Santa Cruz, sc-13073) at 4 °C overnight, and immunoprecipitated with sepharose-Protein G beads (Sigma, P3296) or anti-rabbit IgG beads (Rockland, 00-8800-25), respectively. Seven hundred micrograms of proteins from each subcellular fraction were used for immunoprecipitation following the same experimental conditions. After washing with IP lysis buffer, the beads were boiled in Laemmli buffer and centrifuged at 14,000 rpm for 1 min. The proteins were resolved by SDS-PAGE (12% gel) and subjected to western blot analysis as described below.

### Western blot analysis

MIP/ZEO, MIP/SP, or HCT116 cells were seeded in 60 -mm dishes. After 48 h, cells were treated with 5-FU, CPT-11 or tunicamycin at indicated concentration and time periods. Cells were extracted in lysis buffer [1% Triton-X 100, 120 mM NaCl, 50 mM Tris-HCl, pH 7.5] supplemented with 1% proteinase inhibitor cocktail (Sigma) followed by protein quantification with Bradford assay (Bio-Rad). Equal amount of proteins were separated by SDS-PAGE under reducing conditions and electrotransferred onto a PVDF membrane (Millipore). Blots were incubated with primary antibodies in TBST (TBS containing 0.1% Tween-20) overnight at 4 °C. [1:1000 anti-GRP78 (sc-13968, Santa Cruz and cat. 3177 Cell Signaling Technology (CST)); 1:1000 anti-SPARC (AON-5031, Haematologic Technologies Inc.); 1:200 anti-pPERK (cat. 649402, BioLegend); 1:1000 anti-PERK (CST); 1:1000 anti-peIF2α (cat. 3597, CST); 1:1000 anti-eIF2α (sc-11386, Santa Cruz); 1:250 anti-ATF4 (sc-200, Santa Cruz); 1:1000 anti-pIRE1α (ab124945, Abcam); 1:1000 anti-IRE1α (cat. 3294, CST); 1:10,000 anti-β-actin (G043, ABM); 1:1000 anti-calnexin (cat. 2679, CST) and 1:2000 anti-DNA-PK (cat. 4602, CST)]. The blots were then incubated with anti-mouse immunoglobulin (IgG)-HRP (SH023, ABM) or anti-rabbit IgG-HRP (cat. 7074, CST) followed by enhanced chemiluminescence (ECL) detection. For immunoprecipitation analysis, conformation-specific secondary antibodies were used [1: 5000 anti-mouse IgG-HRP (Rockland) and 1:5000 anti-rabbit IgG-HRP (Rockland)].

### RNA interference

HCT116 cells were plated in six-well plate at 80% confluency. After 24 h, cells were transfected with iLenti-GFP SPARC siRNA or the scramble control siRNA (ABM) using Lipofectamine 2000 (Invitrogen). Cells were selected with puromycin (1 μg/ml).

### Cell-viability assay

Cells were seeded at a density of 4 × 10^3^ per well in 96-well plates. After 24 h, cells were treated with drugs of various concentration for 48 h. (3-(4,5-dimethylthiazol-2-yl)-5-(3-carboxymethoxyphenyl)-2-(4-sulfophenyl)-2H-tetrazolium, inner salt) (MTS) assay using a test kit (Promega) were performed according to the manufacturer’s instruction.

### TUNEL assay

Cells were treated with 5FU (5 μM) and Tm (0.5 μg/ml) for 48 h. The suspension and attached cells were harvested, and fixed onto glass slides with Shandon cytospin at 2000 rpm for 10 min, and stained with in In Situ Cell Death Detection kit (Promega). The number of TUNEL-positive cells was counted and averaged from four independent experiments.

### Immunofluorescence staining and confocal Analysis

Cells were fixed with 4% paraformaldehyde at room temperature (RT) for 15 min followed by incubation with blocking buffer (PBS with 5% NCS, 0.2% Triton X-100) for 1 h at RT andprimary antibodies at 4 °C overnight. The proteins were detected with Alexa 488-Goat anti-mouse IgG, Alexa 647 goat anti-mouse IgG or Alexa 555-goat anti-rabbit IgG (Invitrogen) for 1 h at RT, followed by nucleus staining with Hochest33258. Images were captured on a Leica TCS SP5 II confocal microscope (Leica Microsystems) with ×100 oil objective lenses and numeric aperture of 1.40 N. Images of the cells were acquired from a 0.13-μm optical section, and no labeling was observed when using the secondary antibody alone. Each 3D stack image was deconvolved using Huygens Professional software (version 15.05) (Scientific Volume Imaging) followed by colocalization analysis. Quantitative assessment of colocalization between SPARC and GRP78 was performed by JACoP plugin in ImageJ (NIH), as described^46^. ImageJ colocalization plugin was used to identify and highlight the colocalizing pixels in white. Colocalization Finder plugin was also used to highlight the colocalizing pixels of SPARC and GRP78 on ER-tracker staining image. The percentage of colocalizing signals on ER was calculated based on the following formula: (area of the highlighted regions in which the average ER tracker staining intensity is above the selected threshold)/ (total area of the highlighted regions) x 100%. Intensity threshold was selected to minimize the nuclear regions while including the majority of the ER regions.

### Tissue microarray (TMA) construction and immunohistochemistry

A TMA representing 143 surgically resected colorectal neoplasias was constructed by obtaining two formalin fixed, paraffin embedded cores from representative areas of primary tumors from each patient. In all, 4 -μm-thick sections from the TMA block were deparaffinized in xylene and rehydrated. Sections were then heated in citrate buffer for 15 min for antigen retrieval. Endogenous peroxidase activity was blocked using 0.3% H_2_O_2_ and washed with PBS for 10 min. Immunohistochemical staining with primary antibody against SPARC was carried out using Ultravision LP detection kit (Thermo Fisher Scientific, Fremont, CA, USA). Sections were treated with Ultra V Block for 5 min to prevent nonspecific reaction with primary antibodies, then incubated at 4 °C for 24 h with primary antibodies, followed by incubation with a primary antibody enhancer for 10 min at room temperature. Subsequently, sections were treated with HRP polymer for 15 min and the reaction product was developed using 3,3-diaminobenzidine tetrahydrochloride (Zymed, South San Francisco, CA, USA). The sections were counterstained with hematoxylin and mounted with Tissue-Tek Glas 6419 (Sakura Finetek, Torrance, CA, USA). Negative controls consisted of omission of the primary antibodies. Staining expression scores were based on the number of tumor cells with positive staining in the cytoplasm, and were categorized as follow: 0 or none (expression <10%), 1+ or weak (10–50%), 2+ or strong (50–80%), and 3+ or intense (>80%), by two independent pathologists who were blinded to clinicopathological data. The two expression scores per sample were averaged, with the average representing the patient’s final expression intensity.

### Statistics

Kaplan–Meier method and Cox regression model were used for univariate survival analysis. Kaplan–Meier Method was used to estimate the survival functions, and median survival times and their 95% confidence intervals. The hazard ratios and their 95% confidence intervals were obtained using Cox regression. The independent variables included the staining intensity of GRP78 and SPARC. Conditional inference trees were used to group those ordinal variables as binary variables. Independent variables were then correlated to overall survival and distant relapse-free survival.

## Supplementary information

Supplementary Data
