# Supplementary material for: The interaction between SPARC and GRP78 interferes with ER stress signaling and potentiates apoptosis via PERK/eIF2α and IRE1α/XBP-1 in colorectal cancer
Source: Cell Death Dis. 2019 Jun 26;10(7):504. doi: 10.1038/s41419-019-1687-x (PMC6594974; doi:10.1038/s41419-019-1687-x)
Supplement: Supplementary file 1 — Supplementary Data [file 41419_2019_1687_MOESM1_ESM.pdf]

Supplementary Data Figure 1

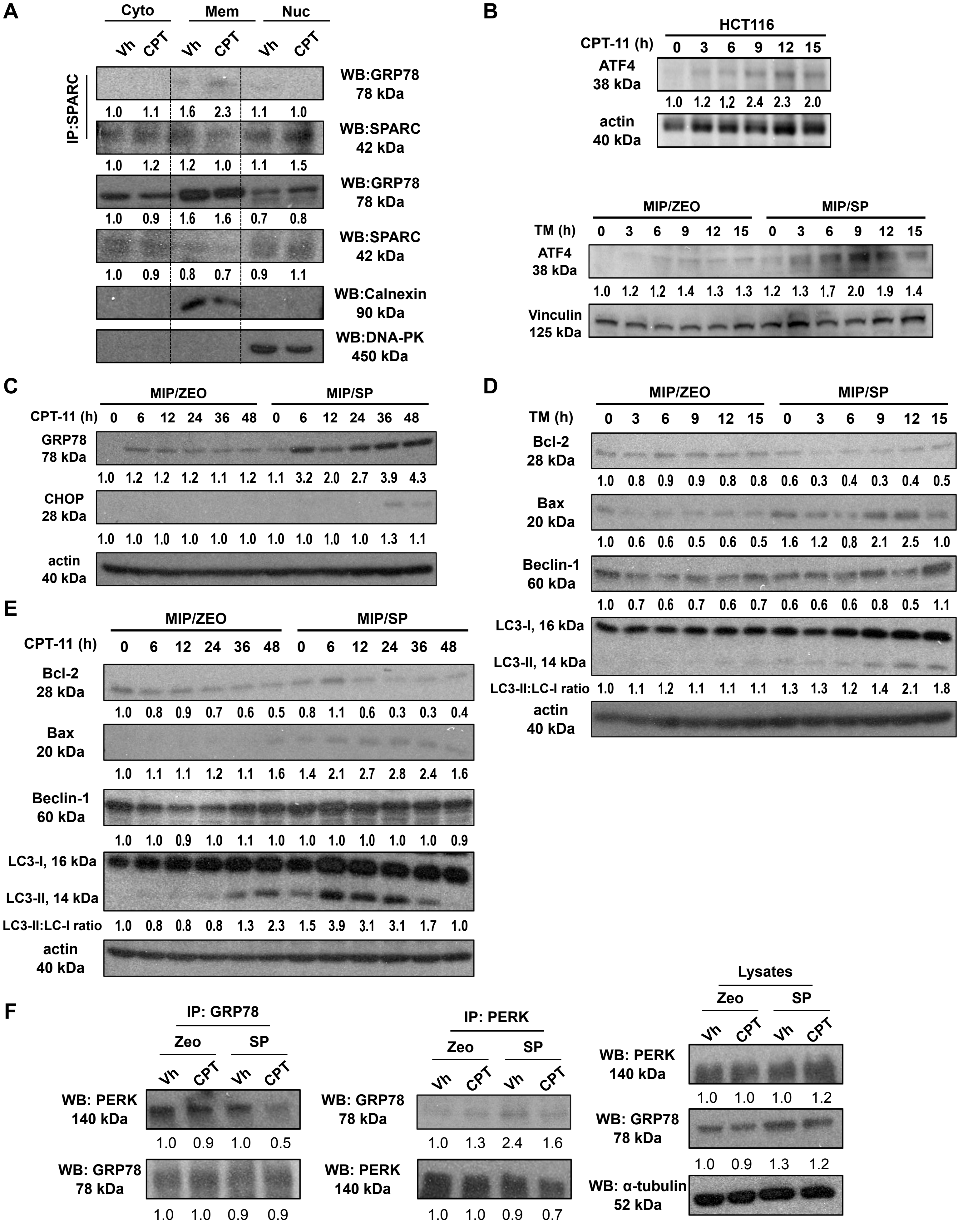

Supplementary Figure 1. Tunicamycin (TM)- and CPT-11-induced ER stress and apoptosis in CRC cells overexpressing SPARC. (A) HCT116 were treated with CPT-11 (50μM, 12h) followed by cell fractionation, co-IP and Western blot analysis of Cyt, Mem and Nuc fractions. Calnexin and DNA-PK served as quality control of cell fractionation. (B) ATF4 induction in HCT116 cells treated with CPT-11 (50μM) and MIP/SP cells treated to 1μg/ml TM were examined by Western blot analysis. (C) Western blot analysis of CHOP induction in CPT-11-treated MIP/SP cells. GRP78 expression confirms persistent ER stress in MIP/SP cells exposed to CPT-11. Apoptosis and Autophagy markers were examined in MIP/Zeo and MIP/SP under (D) TM (1μg/ml) or (E) CPT-11 (50μM) treatment. (F) MIP/Zeo and MIP/SP cells were treated with CPT-11 (50μM, 12 h) and the cell lysates were immunoprecipitated with anti-GRP78 and anti-PERK antibodies, respectively, followed by Western blot analysis.

Supplementary Data Figure 2

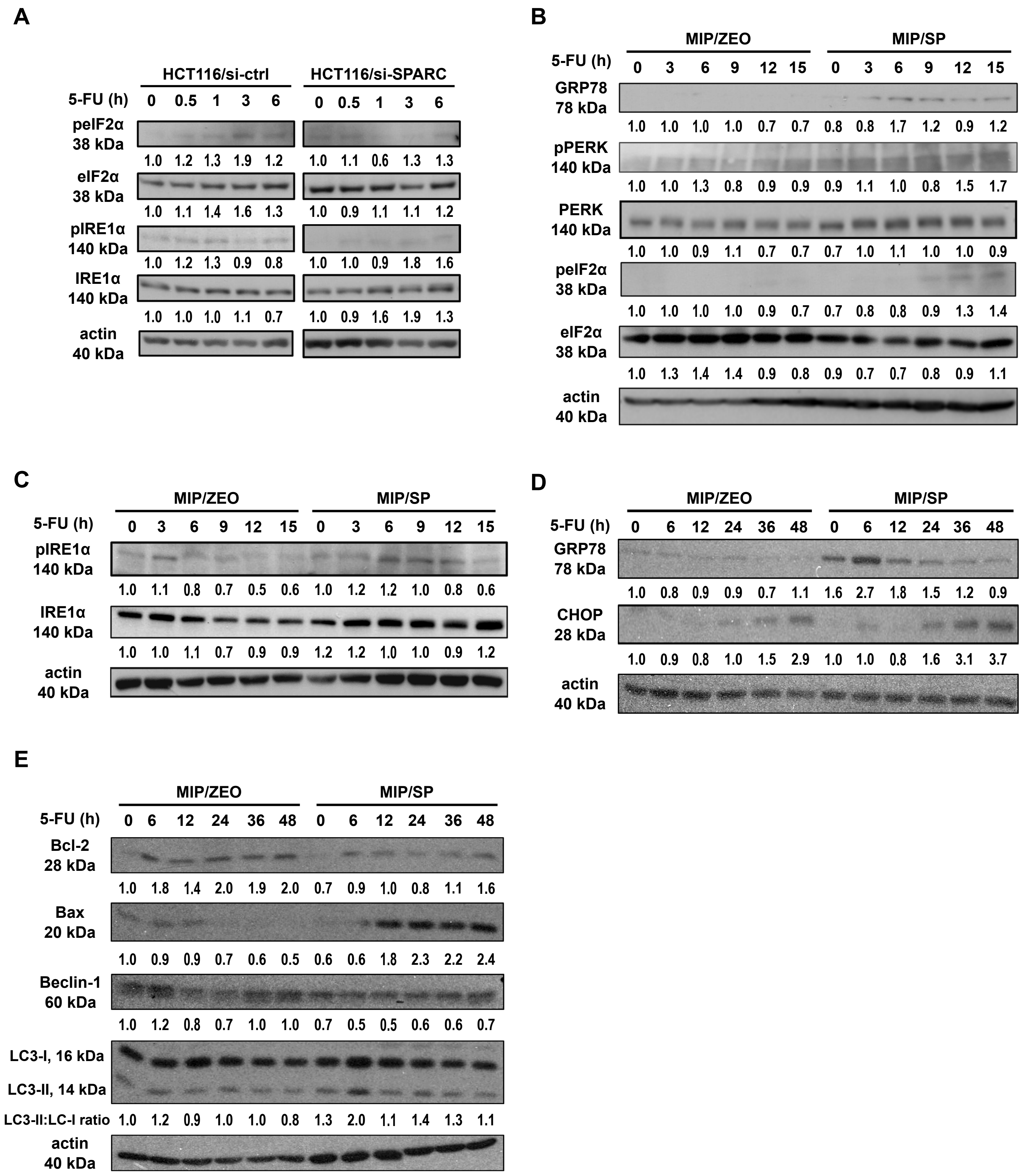

Supplementary Figure 2. 5-FU-induced ER stress and apoptosis in HCT116 and MIP/SP. (A) Western blot analysis showing activation of ER stress signaling in HCT116 cells following SPARC siRNA knockdown and subsequent exposure to 5-FU (25μM). Activation of (B) PERK/eIF2α and (C) IRE1α/XBP-1 signaling in MIP/Zeo and MIP/SP treated with 5-FU (25μM) were examined by Western blot analysis. (D) CHOP induction and (E) expression of apoptosis and autophagy markers were examined in 5-FU-treated MIP/Zeo and MIP/SP cells by Western blot analysis.
